# Correcting names of bacteria deposited in National Microbial Repositories: an analysed sequence data necessary for taxonomic re-categorization of misclassified bacteria-ONE example, genus *Lysinibacillus*

**DOI:** 10.1016/j.dib.2017.06.042

**Published:** 2017-07-05

**Authors:** Bhagwan N. Rekadwad, Juan M. Gonzalez

**Affiliations:** a National Centre for Microbial Resource, National Centre for Cell Science, Pune, India; b School of Life Sciences, SRTM University, Nanded, India; c Institute of Natural Resources and Agrobiology, Spanish National Research Council, IRNAS-CSIC, Avda. Reina Mercedes 10, 41012 Sevilla, Spain

**Keywords:** 16S rRNA, Bacteria, Culture collection, DDH, Digitalization

## Abstract

A report on 16S rRNA gene sequence re-analysis and digitalization is presented using *Lysinibacillus* species (one example) deposited in National Microbial Repositories in India. *Lysinibacillus* species 16S rRNA gene sequences were digitalized to provide quick response (QR) codes, Chaose Game Representation (CGR) and Frequency of Chaose Game Representation (FCGR). GC percentage, phylogenetic analysis, and principal component analysis (PCA) are tools used for the differentiation and reclassification of the strains under investigation. The seven reasons supporting the statements made by us as misclassified Lysinibacillus species deposited in National Microbial Depositories are given in this paper. Based on seven reasons, bacteria deposited in National Microbial Repositories such as *Lysinibacillus* and many other needs reanalyses for their exact identity. Leaves of identity with type strains of related species shows difference 2 to 8 % suggesting that reclassification is needed to correctly assign species names to the analyzed *Lysinibacillus* strains available in National Microbial Repositories.

**Specifications Table**

**Table:** 

Subject area	*Microbiology*
More specific subject area	*Basic Microbiology*
Type of data	*Figure and Tables*
How data was acquired	*Through 16S rRNA sequence analysis and freeware*
Data format	*Raw and analyzed*
Experimental factors	*Not applicable*
Experimental features	*All analysis carried out for bacterial sequences using standard parameters*
Data source location and analysis	*All data analysis was carried out at the School of Life Sciences, S. R. T. M. University, Nanded (India) during 2016.*
Data accessibility	*Data is incorporated within this article*

**Value of the data**

• Generated datasets are useful for visual interpretation and comparative analyses.
• Data act as limelight for differentiation and reclassification of individual species.
• Data give exact visual distribution, thorough analysis of each base pair and the relevance for strain differentiation and prerequisite for classification.

## 1 Data

Data analysis was started in early 2016. *Lysinibacillus* species 16S rRNA gene sequence accession number were picked from respective Microbial Repositories web catalogue. 16S rRNA gene sequences of Lisinibacillus species were downloaded from NCBI website ( https://www.ncbi.nlm.nih.gov/nuccore) from January-May in the year 2016.

**Fig. 1:**
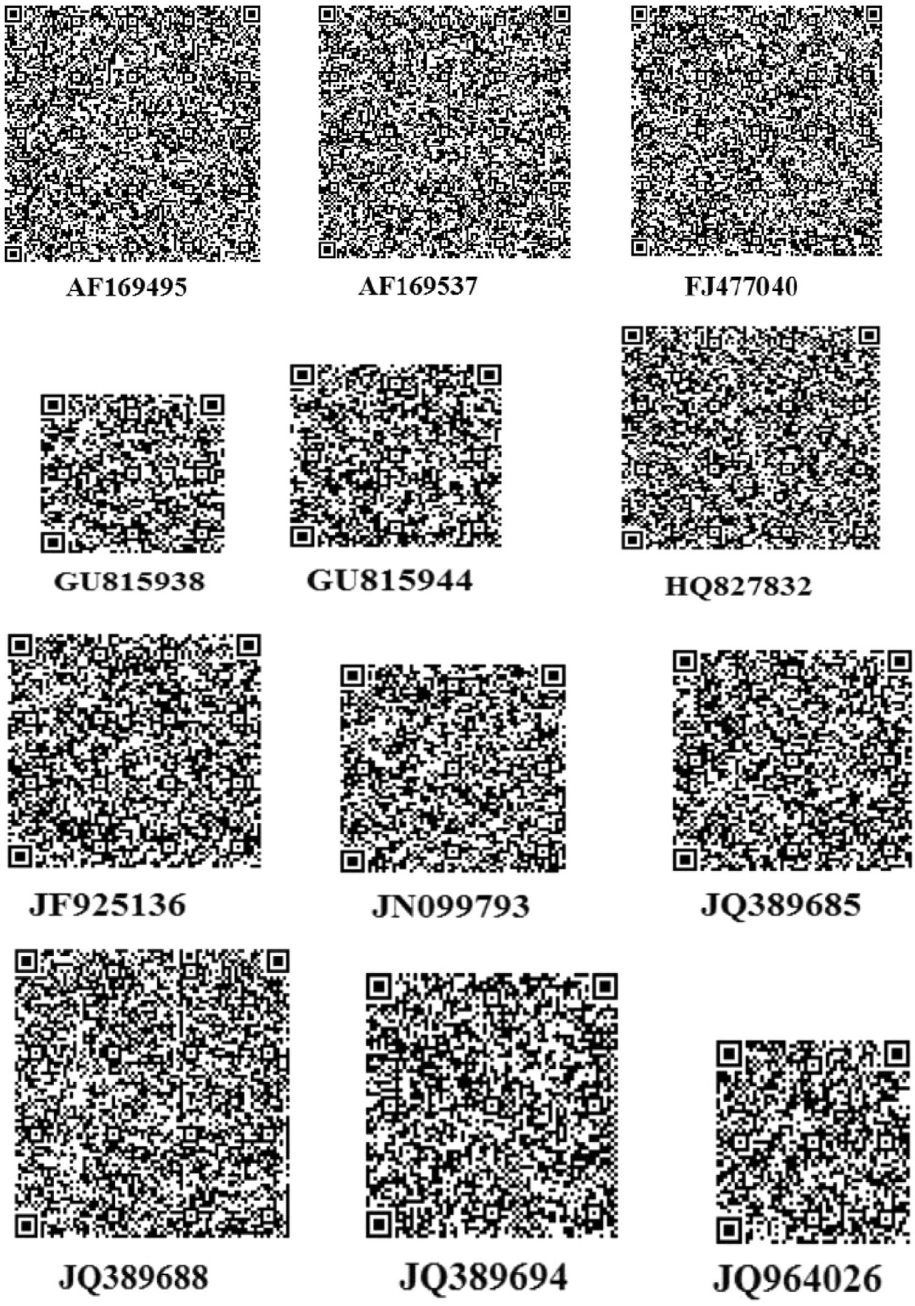

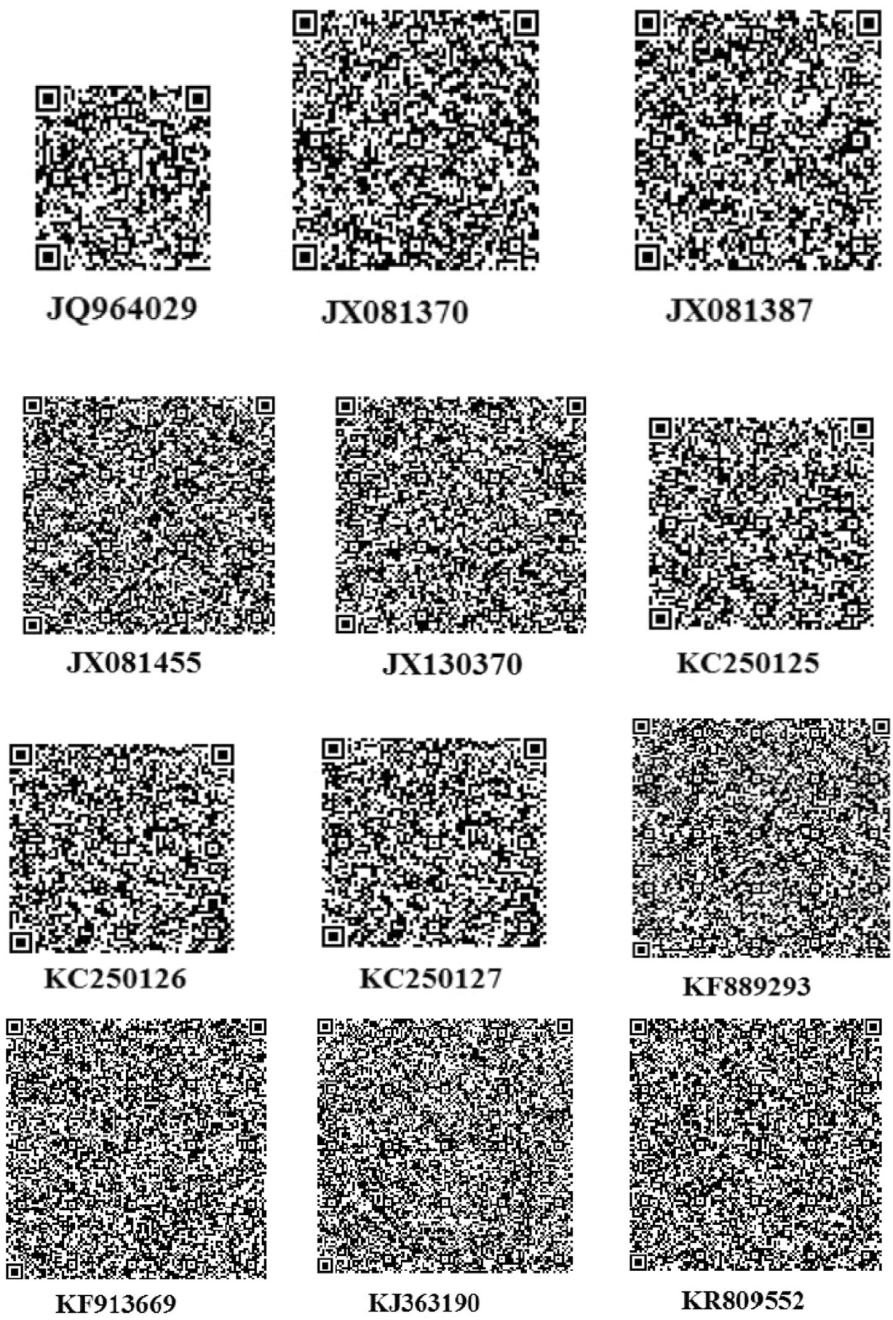
Quick response (QR) codes of *Lysinibacillus strains*.

**Fig. 2:**
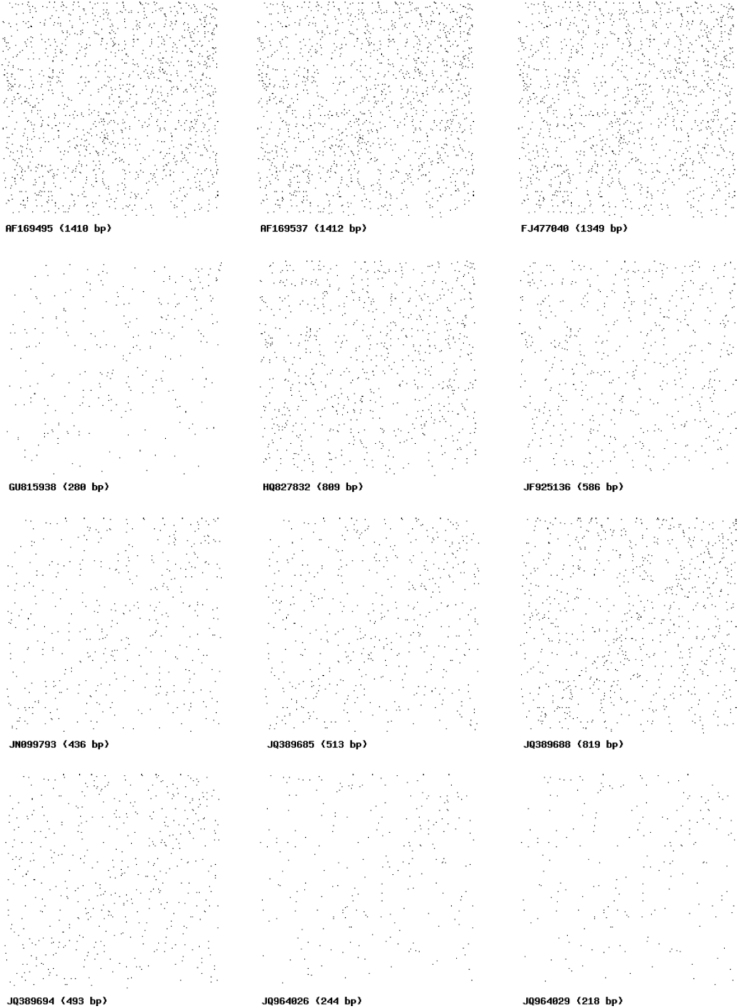

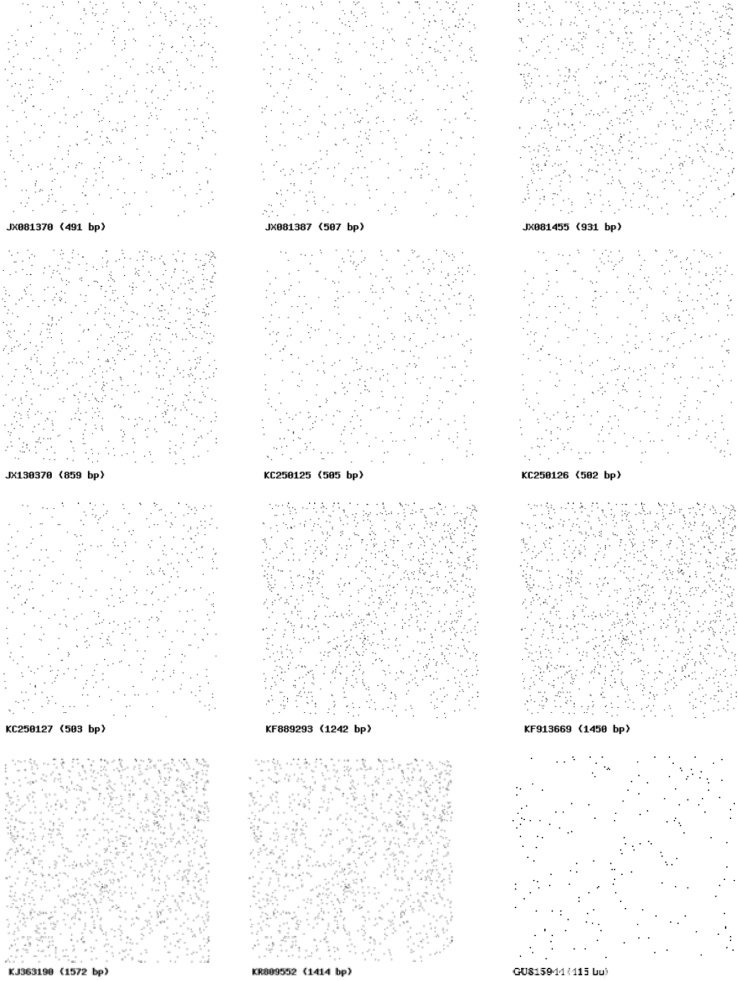
Chaose Game Representation (CGR) of *Lysinibacillus* strains.

**Fig. 3:**
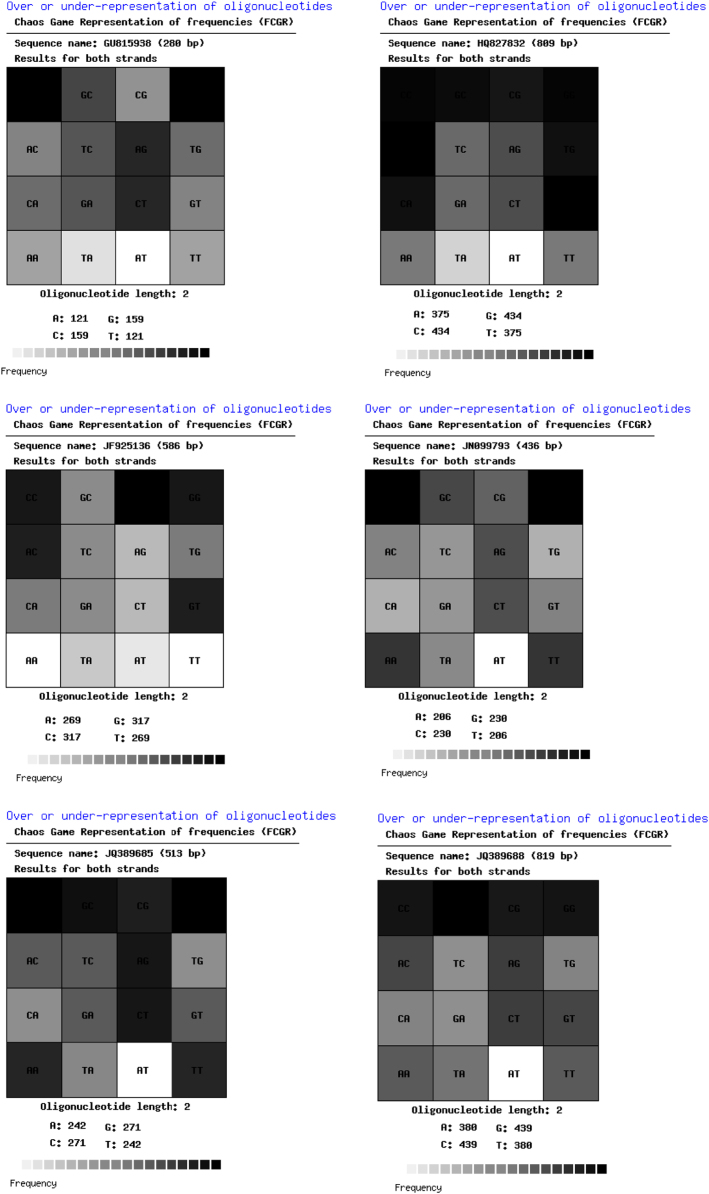

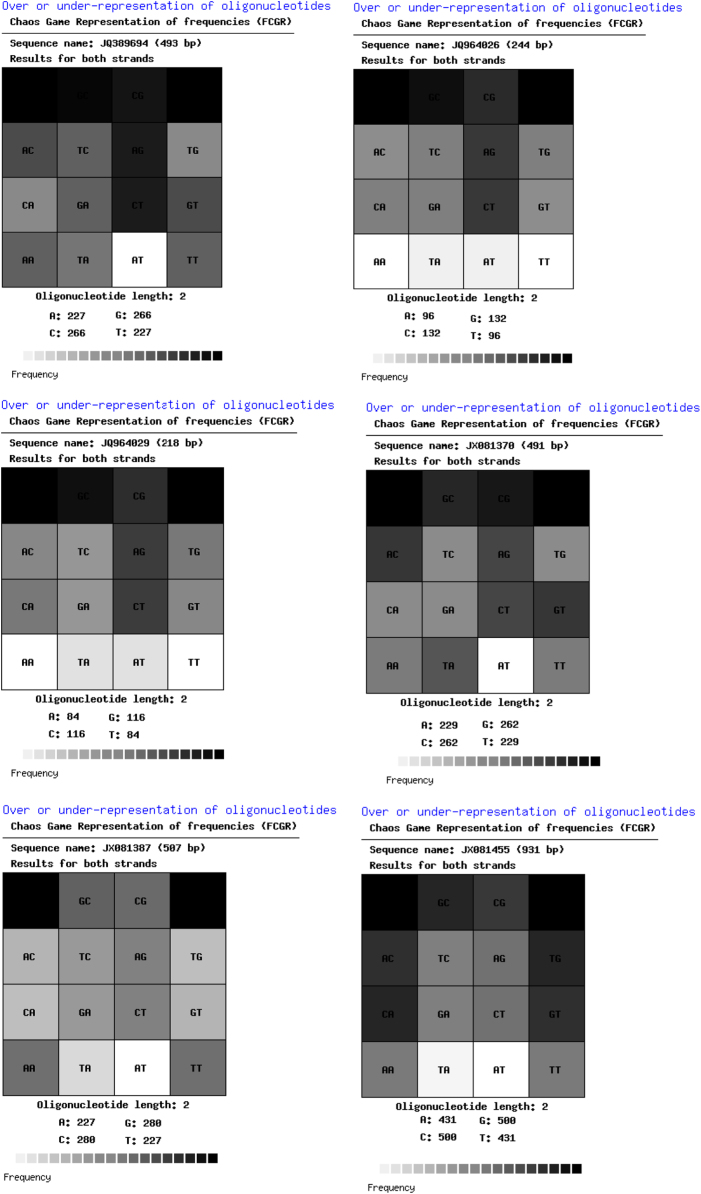

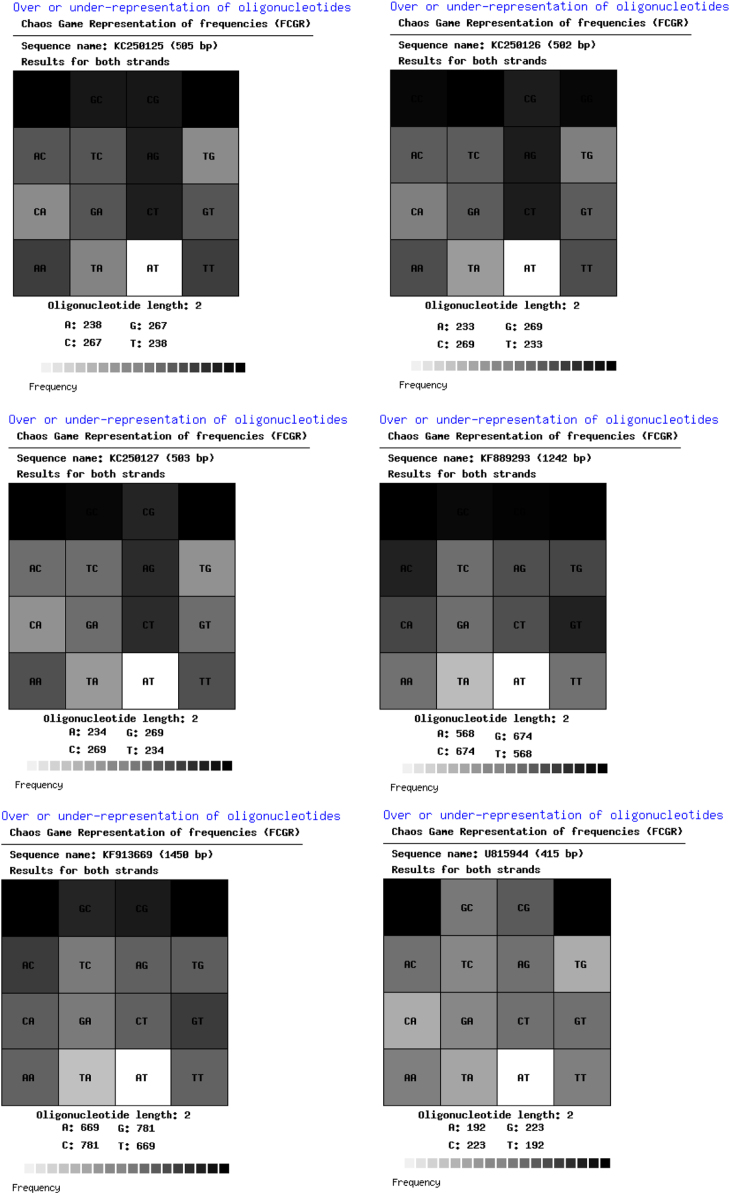
Frequency of Chaose Game Representation (FCGR) for *Lysinibacillus* strains.

**Fig. 4:**
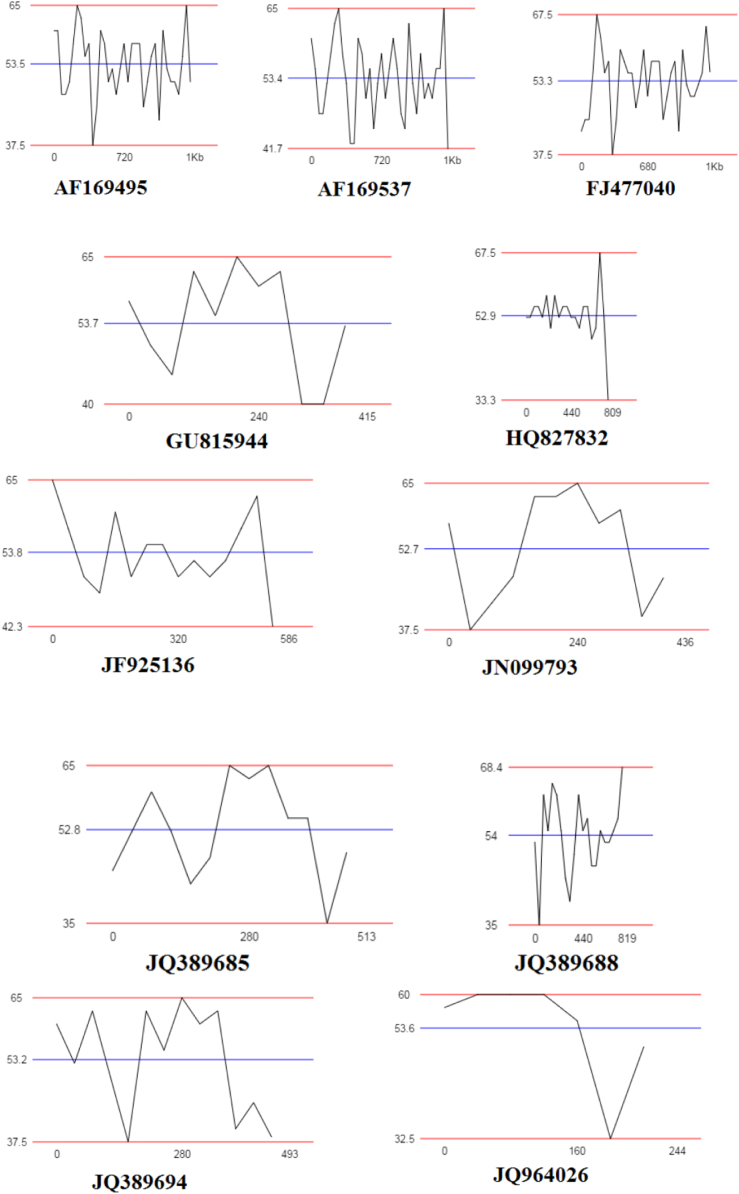

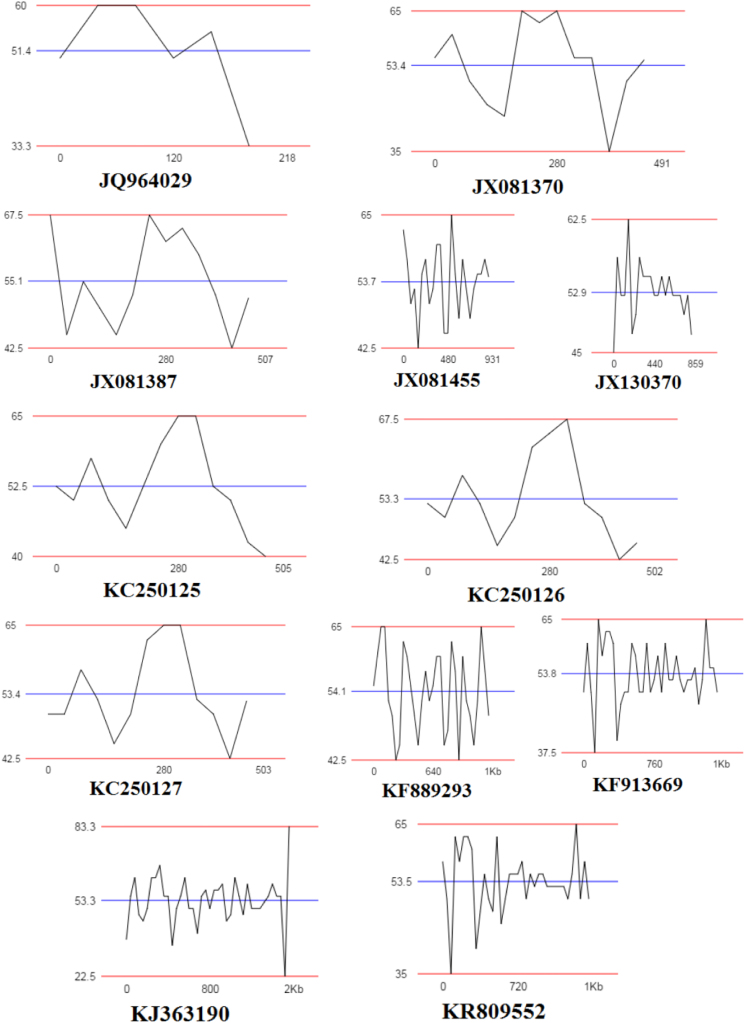
GC plots of *Lysinibacillus* strains based on their 16S rRNA gene sequences.

**Fig. 5:**
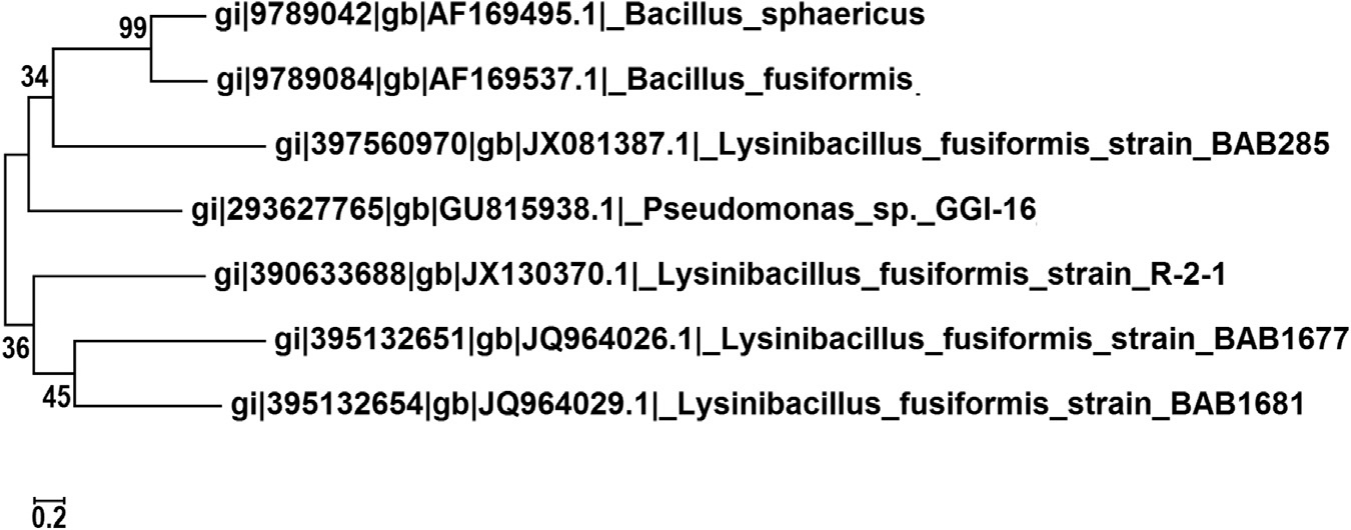
Evolutionary relationships amongst the evaluated *Lysinibacillus* species and type strains from related species showing two lineages and the differentiation of distinct strains.

**Fig. 6:**
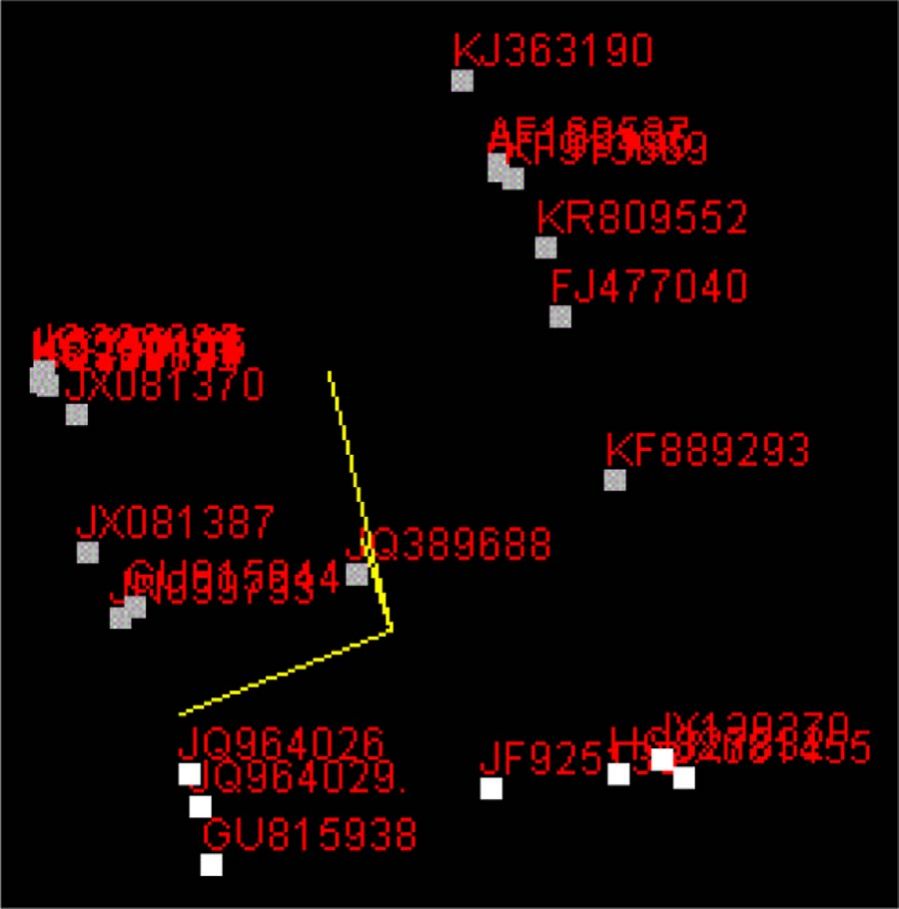
Principle component analysis (PCA) of *Lysinibacillus* strains.

The thoroughly investigated dataset of this article provides information on the misclassified and misplaced bacteria in the microbial culture collections/repositories in India. Fig. 1, Fig. 2, Fig. 3, Fig. 4, Fig. 5, Fig. 6 and Table 1, Table 2, Table 3, Table 4, Table 5, Table 6 explain datasets of the misclassified bacteria.

**Table 1:** *Lysinibacillus* species from National Microbial Repositories in India and their 16S rRNA gene sequences.

**Culture collection**	**Accession number**^a^	**Assigned species**	**Nucleotide length**^a^
Gujarat Biodiversity Gene Bank, Gujarat State Biotechnology Mission (GSBTM), Gandhinagar, India	GU815938	*Lysinibacillus sphaericus*	280
	GU815944	*Lysinibacillus fusiformis*	415
	HQ827832	*Lysinibacillus fusiformis*	809
	JF925136	*Lysinibacillus fusiformis*	586
	JN099793	*Lysinibacillus fusiformis*	436
	JQ389685	*Lysinibacillus fusiformis*	513
	JQ389688	*Lysinibacillus fusiformis*	819
	JQ389694	*Lysinibacillus fusiformis*	493
	JQ964026	*Lysinibacillus fusiformis*	244
	JQ964029	*Lysinibacillus fusiformis*	218
	JX081370	*Lysinibacillus sphaericus*	491
	JX081387	*Lysinibacillus fusiformis*	507
	JX081455	*Lysinibacillus fusiformis*	931
	KC250125	*Lysinibacillus fusiformis*	505
	KC250126	*Lysinibacillus fusiformis*	502
	KC250127	*Lysinibacillus fusiformis*	503
	KF889293	*Lysinibacillus fusiformis*	1242
	KF913669	*Lysinibacillus xylanilyticus*	1450

National Centre for Microbial Resource, National Centre for Cell Science, Pune, India	AF169495	*Lysinibacillus sphaericus*	1410
	AF169537	*Lysinibacillus fusiformis*	1412
	FJ477040	*Lysinibacillus xylanilyticus strain XDB9*	1349
	JX130370	*Lysinibacillus fusiformis strain R-2-1*	859
	KR809552	*Lysinibacillus sphaericus strain S2R3C4*	1414

National Collection of Industrial Microorganisms (NCIM), National Chemical Laboratory (NCL), Pune, India	KJ363190	*Lysinibacillus sp. IT4(2011)*	1572

a 16S rRNA gene sequences.

**Table 2:** *Lysinibacillus* strains from culture collections compared by using GC calculation tool.

**Accession number**^a^	**Nucleotide length**^a^	**GC percentage**		
		**Maximum**	**Minimum**	**Average**
**GU815938**	**280**	**60**	**50**	**56.8**
GU815944	415	65	40	53.7
HQ827832	809	67.5	33.3	52.9
JF925136	586	65	42.3	53.8
JN099793	436	65	37.5	52.7
JQ389685	513	65	35	52
JQ389688	819	68.4	35	54
JQ389694	493	65	37.5	53.2
**JQ964026**	**244**	**60**	**32.5**	**53.6**
**JQ964029**	**218**	**60**	**33.3**	**51.4**
**JX081370**	491	65	36	53.4
JX081387	**502**	**67.5**	**42.5**	**56.1**
JX081455	931	65	42.5	53.7
KC250125	505	65	40	52.5
KC250126	502	67.5	42.5	53.3
KC250127	503	65	42.5	53.4
KF889293	1242	65	42.5	54.1
KF913669	1450	65	37.5	53.8
AF169495	1410	65	37.5	53.5
AF169537	1412	65	41.7	53.4
FJ477040	1349	67.5	37.5	53.3
JX130370	859	65	36	53.4
KR809552	1414	65	35	53.5
**KJ363190**	**1572**	**83.3**	**22.5**	**53.3**

a 16S rRNA gene sequence.

**Table 3:** NCBI-BLAST Analysis report: *Lysinibacillus* sp.

**SL**	**Accession Number**	**% similarity with strain**	**Identity**
1	JQ964026	*Lysinibacillus macroides* strain Se2 (KX959975)	93%
2	JQ964029	*Lysinibacillus fusiformis* (KX397625)	92%
3	JX081387	*Lysinibacillus* sp. 20088723339 (KT254135)	90%
4	GU815938	Bacterium enrichment culture clone ALO1 (JF687759)	90%

**Table 4:** GC content difference between *Lysinibacillus fusiformis*strains and the type strain for this species.

**Accession number**	**Strain**	**Difference of % GC**
AF169537	*Lysinibacillus fusiformis*	16.34
GU815944	*Lysinibacillus fusiformis*	16.42
HQ827832	*Lysinibacillus fusiformis*	16.33
JF925136	*Lysinibacillus fusiformis*	16.78
JN099793	*Lysinibacillus fusiformis*	15.44
JQ389685	*Lysinibacillus fusiformis*	15.51
JQ389694	*Lysinibacillus fusiformis*	16.64
**JQ964026**	***Lysinibacillus fusiformis***	**20.86**
**JQ964029**	***Lysinibacillus fusiformis***	**20.69**
**JX081387**	***Lysinibacillus fusiformis***	**17.91**
JX081455	*Lysinibacillus fusiformis*	16.39
KC250125	*Lysinibacillus fusiformis*	15.56
KC250126	*Lysinibacillus fusiformis*	16.27
KC250127	*Lysinibacillus fusiformis*	16.17
KF889293	*Lysinibacillus fusiformis*	16.96
KJ363190	*Lysinibacillus sp. IT4(2011)*	15.42
JX130370	*Lysinibacillus fusiformis strain R-2-1*	16.05

**Table 5:** GC content difference between *Lysinibacillus sphaericus* strains and the type strain for this species.

**Accession number**	**Strain**	**Difference of % GC**
AF169495	*Lysinibacillus sphaericus*	16.23
**GU815938**	***Lysinibacillus sphaericus***	**19.47**
JX081370	*Lysinibacillus sphaericus*	16.05
KR809552	*Lysinibacillus sphaericus strain S2R3C4*	16.22

**Table 6:** GC content difference between *Lysinibacillus xylanilyticus* strains and the type strain for this species.

**Accession number**	**Strain**	**Difference of % GC**
KF913669	*Lysinibacillus xylanilyticus*	**18.31**
FJ477040	*Lysinibacillus xylanilyticus strain XDB9*	**17.75**
JQ389688	*Lysinibacillus sp.*	**18.05**

**Table 7:** Output of sequence data on EzBioCloud's Identify service (http://www.ezbiocloud.net/identify) database supporting our finding.

Table 7 Output of sequence data on EzBioCloud׳s Identify service (http://www.ezbiocloud.net/identify) database supporting our finding paper is tabulated.

## 2 Experimental design, materials and methods

Twenty-four *Lysinibacillus* strains deposited in renowned microbial culture collections in India were used as a model case for this study (Table 1).

Of the twenty-four *Lysinibacillus* species, eighteen species were from Microbial Repository (Biogene), Gujarat Biodiversity Gene Bank, Gujarat State Biotechnology Mission, Gandhinagar, Gujarat, Five species from National Centre for Microbial Resource, National Centre for Cell Science, Pune. One species was deposited in National Collection of Industrial Microorganisms, National Chemical Laboratory, Pune in India. No *Lysinibacillus* species was found in MTCC-IMTECH, Chandigarh, India. The 16 rRNA gene sequences of these strains were retrieved from the international repositories (https://www.ncbi.nlm.nih.gov/nuccore/) in FASTA format. FASTA rRNA gene sequences of *Lysinibacillus* species were used to generate QR codes, CGR, FCGR, GC percent determination, phylogenetic analysis, principal component analysis [3], [4], [5] and DNA–DNA Hybridization [6]. QR codes were prepared using DNABarID tool (http://www.neeri.res.in/DNA_BarID/DNA_BarID.htm). CGR, FCGR and GC plot were drawn using web-based tools [7], [8]. The phylogenetic tree was constructed using MEGA6.2 tool [9], [10], [11]. PCA was carried out using a multiple alignment program EMBL-EBI MUSCLE [12], [13], [14].

## 3 Background

At present, the 16S rRNA genes are the key for the taxonomic categorization of Bacteria and Archaea. This is due to the existence of extensive sequence information on 16S rRNA genes in public repositories [1] and well curated databases [2]. Nevertheless, the identification of unknown or newly sequenced strains involves comparison with these databases and often a subjective and/or ambiguous set when differentiating novel strains by their 16S rRNA gene sequence. For instance, some 16S rRNA gene sequences are too short limiting the information that can be extracted for comparison and identification. Thus, the accurate identification or classification of strains needs a simple and quick pipeline besides more advanced procedures involving polyphasic approaches (including phenotypic and genomic techniques) for the definitive classification of species. The aim in microbial strain identification and differentiation is to have an available pipeline for unambiguous classification. This paper describes new types of analyses for strain differentiation based on sequence analyses which are easy to perform.

## 4 Results

QR codes prepared from 16S rDNA sequences of *Lysinibacillus* species were unique. Any user can scan QR code using a smart phone and retrieve the sequence (Fig. 1).

CGR and FCGR were used for visual interpretation of the appearance of nucleotides in 16S rRNA genes. Each CGR image has four corners. Upper two corner from left to right were C and T/U, while lower two corners from left to right were A and G. Each CGR square has four sub-squares for nucleotides viz. C, G, A and T/U. A number of dots appeared in sub-square is directly proportional to the number of nucleotides. Distribution of each nucleotide in sub-square indicates the appearance of base pairs in the analyzed gene i.e. sequence, number and percentage (Fig. 2).

Unlike CGR, FCGR presents a different type of visual datasets. Distribution of nucleotides in these matrices is diverse among the studied strains. The FCGR scale indicates from poorly represented dinucleotides (white or light colored) to frequently observed dinucleotides (darkest squares) (Fig. 3).

The nucleotide sequences from JQ964026, JQ964029, JX081387, GU815938 and JX081370 showed high GC percent about 60–67.5% while KJ363190 have 83.3% GC content (Table 2, Fig. 4).

The BLAST analysis of JQ964026, JQ964029, JX081387 and GU815938 sequences showed 93%, 92%, 90% and 90% identity with existing species and type strains. This was confirmed from phylogenetic analysis, principal component analysis and GGDC-DDH results. The phylogenetic tree was constructed including Lysinibacillus and phylogenetically related species with bootstrap values corresponding to 1000 replicates (Fig. 5).

The 16S rRNA gene sequences JQ964026, JQ964029, JX081387 and GU815938 showed identities lower than 97% (90–93% with existing species and type strains) (Table 3) suggesting that they could potentially belong to different species. Table 3 suggests a clear distinction between *Lysinibacillus* strains below the expected level for species differentiation.

Results of Principal Component Analysis comparing the 16S rRNA gene sequences (Fig. 6) revealed different groups which could be related to major novel species or taxa within the Lysinibacillus genus.

Most of these strains were isolated from environmental samples such as boron containing soil, forest humus collected from Gyeryong Mountain in Korea, Environmental Treatment Plant Naroda G.I.D.C., Ahmedabad, Gujarat (India) and textile mill effluent contaminated soil etc., followed by acclimatization on the presence of different chemicals such as Boron, Sodium Chloride, Xylan, dyes etc [15], [16], [17], [18]. This information suggests that different adaptations could result in differential strains with distinctive 16S rRNA gene sequences. GGDC-DDH analysis with type strains indicated all species has G+C difference ranged from 15.44 to 20.86 (Table 4, Table 5, Table 6). These analyses suggest that the *Lysinibacillus* strains could represent distinct species deposited in Indian Microbial Repositories. Thus, there is a gap of information on accurate classification within this genus and specifically on this group of strains that have been used as a model case to describe this current identification issue.

**Special note:** The reason for the statements made by us as misclassified Lysinibacillus species deposited in National Microbial Depositories: (a) Erroneous sequences. (b) Mismatch of identity with the top hit taxon on NCBI nucleotide-nucleotide BLAST and EzTaxon database. (c) Very less percentage similarity and less than 92.22-99.0 percent match with Standard type strains. (d) Very less completeness score. (e) Very short 16S rRNA sequences. (f) Very long sequences with chimaeras. (g) Doubtful contigs or single long and unassembled sequence Based on above seven reasons, bacteria deposited in National Microbial Repositories such as Lysinibacillus either need to be re-sequenced for 16S rRNA gene and should be reanalysed on EzBioCloud׳s database for their exact identity or identified using appropriate valid techniques (Table 7).

## 5 Discussion

This study provides a pipeline to structure 16S rRNA gene sequence information constructing digitalized datasets on *Lysinibacillus* strains currently present in several culture collections (GSBTM Gujarat, NCMR-NCCS Pune and NCIM-NCL Pune) in India and many other National Culture Collections in the world. This information contributes to identify, compare, evaluate, interpret strain, species differentiation for novel isolates from environmental samples and make compulsory rule to investigate the correct identity of bacteria with them. Differentiation of bacteria obtained from an environment results in a relatively complicated task when those bacteria are phylogenetically closely related among them. This issue gets enhanced when comparing and classifying bacteria related to poorly curated sequence data and scarcely analyzed strains lacking a fulfillment of polyphasic recommendations. An easy differentiating pipeline represents a greatly useful tool for a large number of applications including species classification of new isolates from natural and artificial environments. The type of digitalized data from this study can be produced for any prokaryotic species and eukaryote sequence data. It could be expanded to the use of genomes or different genes or sets of genes. Overall, the enlisted data and protocol will be useful to research and industry. The proposed pipeline greatly contributes to simplify the identification and differentiation of unclassified strains and the needs for reclassification of some previously isolated microorganisms, including the detection of microbes based on 16 S rRNA gene sequence information from microbial community surveys. The proposed approach can increase its specificity and applicability as needed using different genes or genome sequence information. Thus, this protocol allows the phenotype and genotype characteristic for reintroduction and taxonomic categorization of species in current pipeline.

## Conflicts of interests

The author declares there are no any conflicts of interest.
