# Supplementary material for: Correcting names of bacteria deposited in National Microbial Repositories: an analysed sequence data necessary for taxonomic re-categorization of misclassified bacteria-ONE example, genus Lysinibacillus
Source: Data Brief. 2017 Jul 5;13:761–78. doi: 10.1016/j.dib.2017.06.042 (PMC5520958; doi:10.1016/j.dib.2017.06.042)
Supplement: Supplementary file 1 — Supplementary material [file mmc1.doc]

**Conflict of interests**

Author (s) declares there is not any conflict of interests
